# Screening in larval zebrafish reveals tissue-specific distribution of fifteen fluorescent compounds

**DOI:** 10.1242/dmm.028811

**Published:** 2017-09-01

**Authors:** Yuxiao Yao, Shaoyang Sun, Fei Fei, Jingjing Wang, Youhua Wang, Ranran Zhang, Jing Wu, Lian Liu, Xiuyun Liu, Zhaomeng Cui, Qiang Li, Min Yu, Yongjun Dang, Xu Wang

**Affiliations:** 1 Key Laboratory of Metabolism and Molecular Medicine, Ministry of Education, Department of Biochemistry and Molecular Biology, School of Basic Medical Sciences, Fudan University, Shanghai 200032, China; 2 Longhua Hospital, Shanghai University of Traditional Chinese Medicine, Shanghai 200032, China; 3 Institute of Reproduction and Development, Collaborative Innovation Center of Genetics and Development, Children's Hospital of Fudan University, Shanghai 201102, China; 4 Deparment of Pediatric Endocrinology and Inherited Metabolic Diseases, Children's Hospital of Fudan University, Shanghai 201102, China; 5 Translational Medical Center for Development and Disease, Shanghai Key Laboratory of Birth Defects, Institute of Pediatrics, Children's Hospital of Fudan University, Shanghai 201102, China

**Keywords:** Zebrafish, Drug screening, Bone staining, Hyperplasia, Purpurin, Epirubicin

## Abstract

The zebrafish is a prominent vertebrate model for low-cost *in vivo* whole organism screening. In our recent screening of the distribution patterns of fluorescent compounds in live zebrafish larvae, fifteen compounds with tissue-specific distributions were identified. Several compounds were observed to accumulate in tissues where they were reported to induce side-effects, and compounds with similar structures tended to be enriched in the same tissues, with minor differences. In particular, we found three novel red fluorescent bone-staining dyes: purpurin, lucidin and 3-hydroxy-morindone; purpurin can effectively label bones in both larval and adult zebrafish, as well as in postnatal mice*,* without significantly affecting bone mass and density. Moreover, two structurally similar chemotherapeutic compounds, doxorubicin and epirubicin, were observed to have distinct distribution preferences in zebrafish. Epirubicin maintained a relatively higher concentration in the liver, and performed better in inhibiting hepatic hyperplasia caused by the over-expression of *kras^G12V^*. In total, our study suggests that the transparent zebrafish larvae serve as valuable tools for identifying tissue-specific distributions of fluorescent compounds.

## Abstract (precis)

**Summary:** Fifteen fluorescent compounds have tissue-specific distributions in zebrafish larvae: three anthraquinones were identified as bone dyes in live vertebrates, and two chemotherapeutic drugs were shown to have distinct preferences for the liver.

## INTRODUCTION

Due to their small size, optical transparency, high fecundity and similarity to humans in genetics and anatomy, zebrafish (*Danio rerio*) serve as a novel mainstream vertebrate model with significant advantages in studying developmental genetics and human diseases (Takaki et al., 2012; Asnani and Peterson, 2014; Goessling and Sadler, 2015; Leung and Mourrain, 2016; MacRae and Peterson, 2015). Recently, zebrafish have also become a valuable tool for investigating the novel functions of small-molecule compounds, and provide a platform for high-throughput targeted drug screening (Burns et al., 2005; Delvecchio et al., 2011; Robertson et al., 2014). The larval zebrafish after 5 days post-fertilization (dpf) is a typical vertebrate animal with most vital organs segmented, including the eye, brain, heart, liver, gut and gallbladder (Goessling and Sadler, 2015). Moreover, treatment of zebrafish larvae with phenylthiourea (PTU) inhibits melanization and makes these fish ideal for detecting the dynamic distribution of fluorescent compounds within a live organism (Laughlin et al., 2008).

Fluorescence is one of the intrinsic characteristics of some minerals and a variety of bio-organic molecules including proteins. Small-molecule drugs such as doxorubicin, daunorubicin, menadione, ellipticine and harmalol carry fluorophores, which allow the molecules to be visualized at the subcellular level in cell culture, and thereby indicate the sites of functioning (Huang et al., 2012). For example, the distributions of doxorubicin and daunorubicin in MCF-7 breast cancer cells and glioma stem cells can be clearly observed in the nucleus, where they intercalate with DNA and induce cytotoxicity (Li et al., 2014; Suarasan et al., 2016). The tissue-specific distributions of some strong fluorescent compounds have also been detected *in vivo* at the whole organism level, but it is difficult to capture high-resolution fluorescence images from deep tissues within conventional model animals like the mouse and rat (Shi et al., 2013; Kuchimaru et al., 2016). Thus we employed 6 dpf zebrafish larvae for studying the distribution patterns of selected fluorescent compounds, with the hope of identifying novel tissue-specific dyes suitable for live animals, and to predict the potential applications or side-effects of certain approved drugs.

Fluorescent compounds that have emissions at longer wavelengths in the visible spectrum can provide better tissue penetration, and in turn increase the overall signal-to-noise ratio. Here, we selected 71 small molecules with strong red fluorescence from a library of 3432 small-molecule compounds, and investigated their distributions in 6 dpf zebrafish larvae via direct observation. In total, we identified 15 compounds with specific distribution patterns, including three novel red fluorescent bone dyes: purpurin, lucidin and 3-hydroxy-morindone. Interestingly, purpurin also labelled adult zebrafish and live mouse bones without significantly affecting bone mass and density. Moreover, two chemotherapeutic drugs, doxorubicin and epirubicin, were observed to have different hepatic enrichments, and epirubicin performed better than doxorubicin in inhibiting the proliferation of hepatocytes in a *kras^G12V^* over-expressing hepatic hyperplasia model. Our results suggest that the distribution patterns of most compounds in live zebrafish larvae were consistent with the clinical pharmacokinetics or the results from mammalian models, and screening in zebrafish larvae can provide compatible information about side-effects or potential new uses of fluorescent or fluorescence-labelled compounds in a quick and effective way.

## RESULTS

### Identification of red fluorescent compounds with tissue-specific distributions in zebrafish larvae

**Fig. 1.**
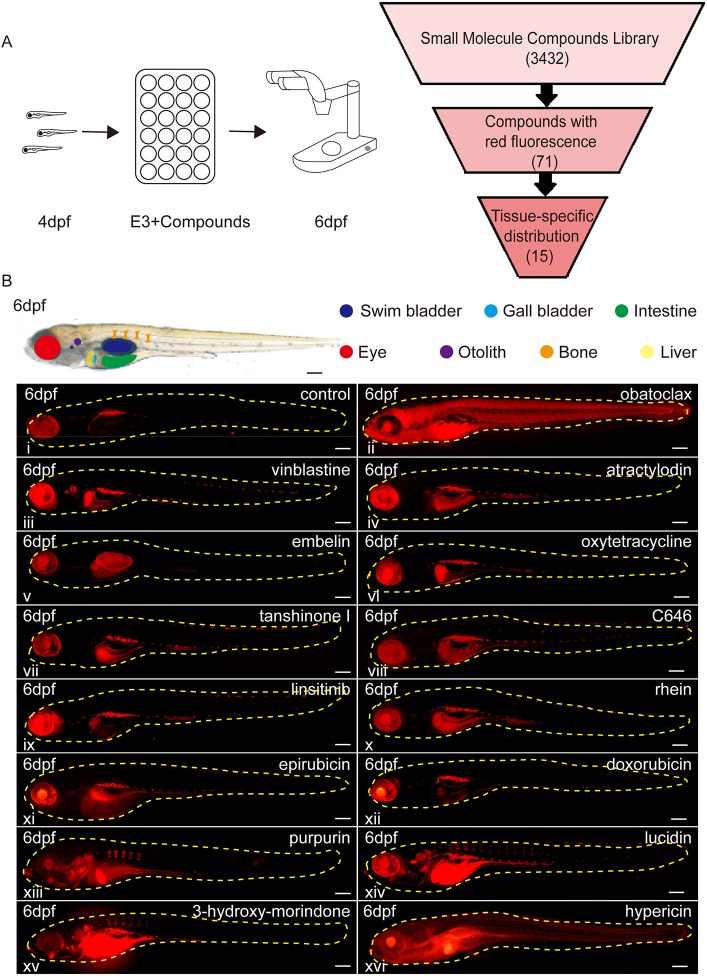
**Identification of red fluorescent compounds with tissue-specific distributions in zebrafish larvae.** (A) An illustration of the screening process. (B) Whole mount lateral views of the zebrafish larvae at 6 dpf with tissue-specific distributions of the red fluorescent compounds. i: DMSO-treated zebrafish as control. Scale bars: 100 μm.

To determine the list of compounds to be used in the screening, we manually identified strong red fluorescent compounds from a library of 3432 small-molecule compounds. A total of 71 compounds were selected as candidates, and they all possessed red fluorescence that could be easily observed at 10–20 mM stock concentrations (Fig. 1A). The CAS numbers, formulas and stock concentrations of those 71 candidates are listed in Table S1. To assess the tissue/organ-specific distributions of the 71 compounds, zebrafish larvae were arrayed into 24-well plates (six larvae per well), and were incubated in E3 medium with 10–20 μM of the fluorescent compounds from 4 dpf to 6 dpf, followed by examination by fluorescent microscopy (Fig. 1A). A total of 15 compounds were observed to have tissue-specific distribution patterns without inducing significant morphological defects in the zebrafish larvae, including vinblastine, atractylodin, tanshinone I, linsitinib, C646, epirubicin, oxtetracycline, purpurin, embelin, obatoclax, rhein, doxorubicin, hypericin, lucidin and 3-hydroxy-morindone (Fig. 1B). The formulas, known applications and distributions of the compounds, as well as the numbers of samples with successful staining, are briefly listed in Table 1. Lateral views of the larvae treated with fluorescent compounds are shown in Fig. 1B; illustrations and structural formulas are provided in Fig. S1. The bright-field images are shown in Fig. S2 and additional images of selected larvae from different views or at distinct stages are shown in Fig. S3.

**Table 1.**
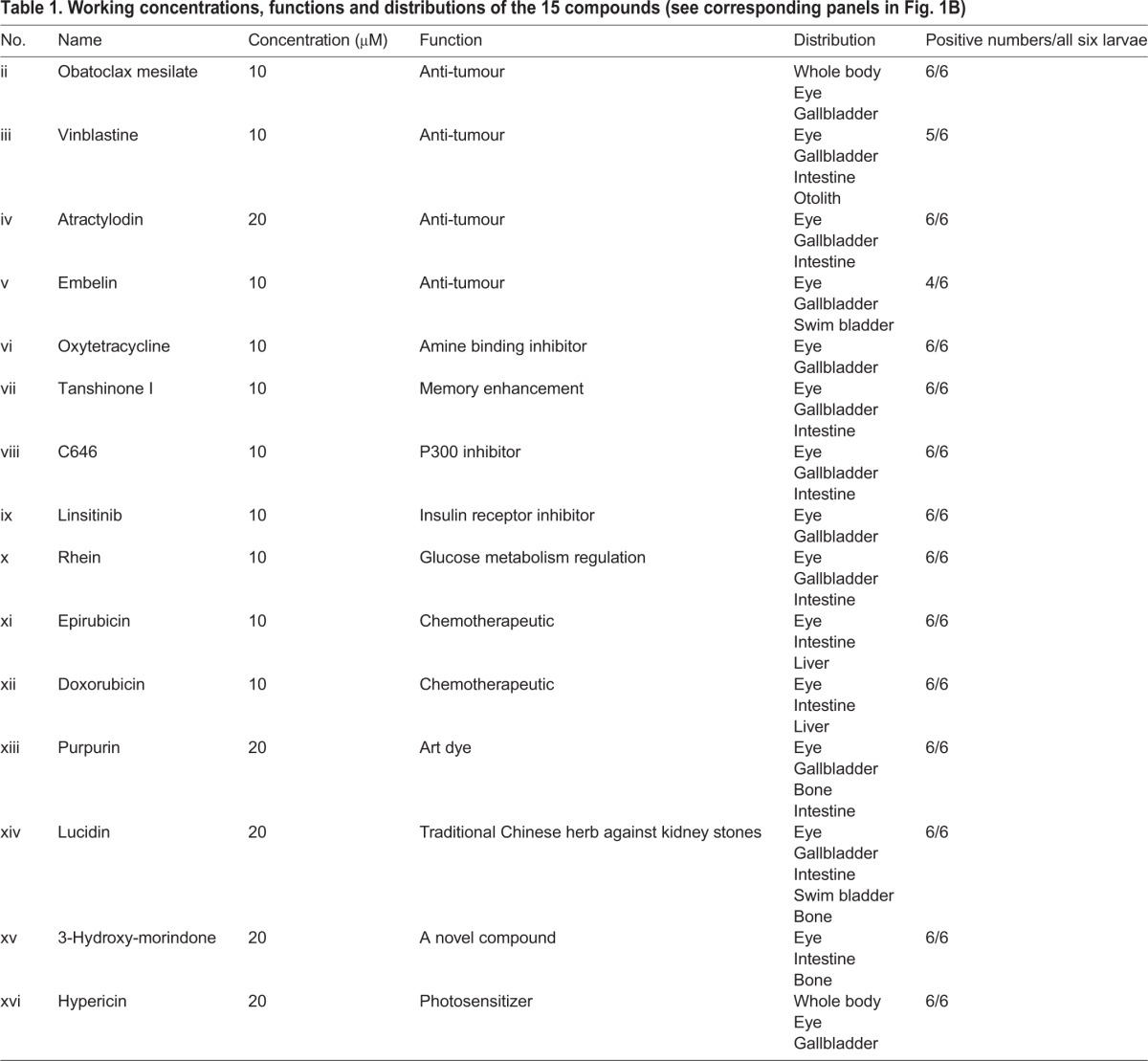
**Working concentrations, functions and distributions of the 15 compounds (see corresponding panels in Fig. 1B)**

Several findings in the tissue-specific distributions of the tested fluorescent compounds predict that they may possess potential side-effects. For example, vinblastine, an alkaloid from *Vinca rosea*, has been used as a classical chemotherapeutic drug for its inhibition of tubulin polymerization into microtubules in highly proliferating cells (Karnofsky, 1964; Zhou and Rahmani, 1992; Lee et al., 2015). Here, we observed that vinblastine accumulated significantly in zebrafish otolith, a biomineralized structure critical for hearing and balance in teleosts, predicting possible damage to the auditory senses (Fig. 1Biii; Fig. S3A) (Stooke-Vaughan et al., 2015). In fact, it is rarely reported that vinblastine may be responsible for ototoxicity in patients receiving multi-component medicine with vinblastine as one of the major ingredients, and the potential deafness risk is not mentioned in all current vinblastine products on the market (Moss et al., 1999). Our observation suggests that the ototoxic drug safety of vinblastine needs to be confirmed and seriously re-evaluated. Moreover, rhein, which is an anthraquinone compound isolated from rhubarb with multiple pharmacological applications in anti-inflammation, anti-tumour, anti-oxidant, anti-fibrosis, hepatoprotective and neuroprotective therapies, was observed to accumulate in zebrafish intestinal lumen (Fig. 1Bx; Fig. S3B) (Sun et al., 2016). Interestingly, rhubarb, the major natural source of rhein, was traditionally used as a purgative in China, and was often used to establish chronic diarrhoea animal models (Qin et al., 2011). Although recent studies suggested that rhubarb extract induced gut microbial diversity in rats, and helped to prevent obesity in mice on a chronic high-fat diet (Peng et al., 2014; Wang et al., 2016), our results suggest that rhein may act in the intestinal lumen and be one of the effective constituents functioning in the intestinal lumen.

The distribution patterns revealed in this study may also help in investigating novel compounds/drugs. For instance, one of the experimental drug candidates for the treatment of various types of cancer is obatoclax, which is a pan-inhibitor of the anti-apoptotic Bcl-2 protein (Konopleva et al., 2008; Yu et al., 2016). The results from a phase I/II study of obatoclax as single agent indicated that obatoclax could not induce an objective response in older patients (i.e. ≥70 years old) with untreated acute myeloid leukaemia at the maximum tolerated dose (Schimmer et al., 2014). In our zebrafish assay, obatoclax was observed to be ubiquitously distributed around the whole larvae, which may explain the multiple side-effects and the limiting factor in raising the dose into an effective level (Fig. 1Bii; Fig. S3E). To further exploit and evaluate obatoclax in clinical trials, using tissue/organ-specific delivery approaches in combination with other chemotherapy modalities may be considered to restrain obatoclax in the targeted tissues.

This study identified several compounds that would be useful dyes to label specific tissues in living animals. In addition to the three bone dyes to be discussed shortly, we also found an interesting compound, embelin, which is a potential fluorescent probe for collagen. Embelin is a natural product isolated from the Japanese *Ardisia* herb, and it exhibits anti-tumour activity through blocking the activity of the X-linked inhibitor of apoptosis protein (Nikolovska-Coleska et al., 2004; Aird et al., 2008; Dai et al., 2009). Previously, embelin was reported to be a cross-linker for both type I collagen through non-polar amino acids (proline, glycine and valine) and type III collagen through the non-polar amino acid, glycine and polar amino acid, lysine, and it can stabilize the collagen structure (Swamy et al., 2011). Interestingly, in 6 dpf larvae, embelin was observed to specifically label the swim bladder, which is a tissue rich in collagen (Fig. 1Bv; Fig. S3C). To further investigate whether embelin can label collagen, adult swim bladders were dissected and stained with a collagen I antibody. Embelin was observed to specifically localize to the collagen I-positive fibroblast cytoplasm in the swim bladder, suggesting that embelin may provide an alternative approach for detecting collagen-enriched tissues in live vertebrates *in vivo* (Fig. S3Civ).

### Three anthraquinones with similar structures label bones *in vivo*

Three red fluorescent compounds were shown to be capable of binding bone in zebrafish larvae: purpurin, lucidin and 3-hydroxy-morindone (Fig. 1Bxiii–xv). Interestingly, these three compounds share similar molecular structures as they all belong to the 9,10-anthraquinone family (Fig. S1), of which Alizarin Red and rhein are also members. Purpurin, lucidin and Alizarin Red were originally extracted from the roots of *Rubia tinctorum* L. (madder root), while purpurin was a colorant used in art works for many years (Melo and Claro, 2010), and lucidin was used as a traditional herbal medicine against kidney stones (Westendorf et al., 1998).

**Fig. 2.**
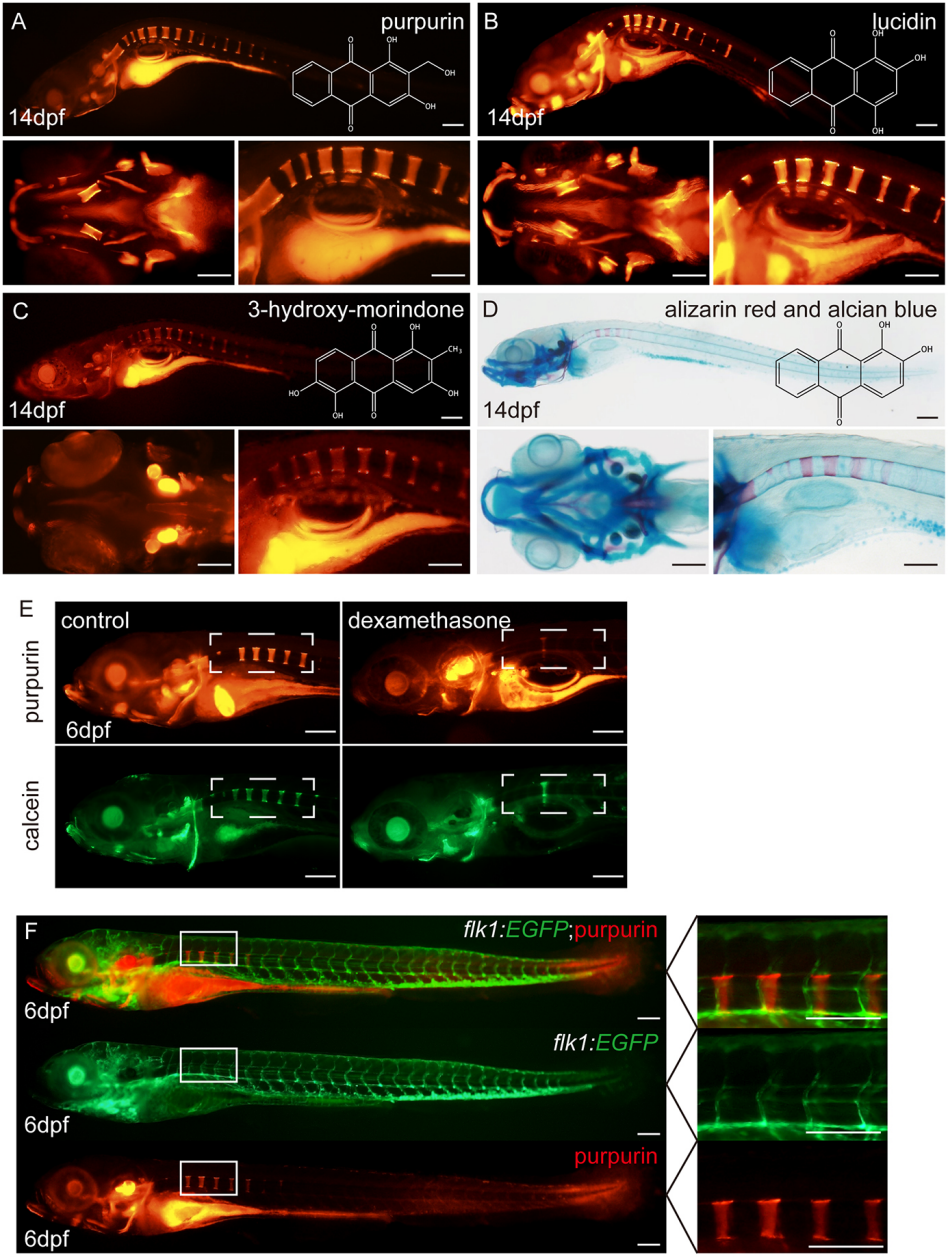
**Purpurin, lucidin and 3-hydroxy-morindone label bones in zebrafish larvae.** (A) Lateral and top views of the purpurin-treated zebrafish larva at 14 dpf, and the chemical structure of purpurin. Lateral view of whole zebrafish (top panel), the top view of the head (lower left panel), and the lateral view of the spine (lower right panel). Scale bars: 100 μm. (B) Lateral and top views of the lucidin-treated zebrafish larva at 14 dpf, and the chemical structure of lucidin. Lateral view of whole zebrafish (top panel), the top view of the head (lower left panel), and the lateral view of the spine (lower right panel). Scale bars: 100 μm. (C) Lateral and top views of the 3-hydroxy-morindone-treated zebrafish larva at 14 dpf, and the chemical structure of 3-hydroxy-morindone. Lateral view of whole zebrafish (top panel), the top view of the head (lower left panel), and the lateral view of the spine (lower right panel). Scale bars: 100 μm. (D) Lateral and top views of the Alizarin Red and Alcian Blue-stained zebrafish larva at 14 dpf. Lateral view of whole zebrafish (top panel), the top view of the head (lower left panel), and the lateral view of the spine (lower right panel). Scale bars: 100 μm. (E) Lateral views of the wild-type and dexamethasone-treated zebrafish larva at 6 dpf. Scale bars: 100 μm. (F) Lateral views of purpurin-treated *Tg(flk1:EGFP)* zebrafish larvae at 6 dpf. Scale bars: 100 μm.

We first repeated the incubation assay in zebrafish from 12 dpf to 14 dpf, and compared the labelling effects of the three anthraquinone compounds with Alizarin Red and Alcian Blue staining, which is the classic staining approach for mineralized bone and cartilage (Walker and Kimmel, 2007). Alizarin Red is known to stain mineralized bone by forming precipitates with free (ionic) calcium (Puchtler et al., 1969), and it seems that these three novel compounds were equally capable of labelling bones in live zebrafish (Fig. 2). Of the three compounds, purpurin and lucidin clearly labelled the mandible and cranium in the zebrafish head, while 3-hydroxy-morindone was poorly localized in those areas (Fig. 2A,B). Moreover, purpurin staining maintained a better signal-to-noise ratio than lucidin, and we therefore chose purpurin to perform the following experiments. We also compared the staining effects of purpurin and calcein on 6 dpf larvae, and the two compounds worked equally well in labelling bone structures (Fig. 2E).

To test whether purpurin can be used to help identify developmental bone defects in zebrafish larvae, we treated 2–6 dpf larvae with 20 μM dexamethasone to interrupt bone calcification as previously reported (Luo et al., 2016). Compared with the control group, dexamethasone-treated larvae displayed severe defects in bone development, with significantly delayed calcification in the anterior vertebral column, which can be clearly distinguished via either purpurin or calcein staining (Fig. 2E). In addition, we also performed purpurin bone staining in *Tg(flk1:EGFP)*, which can mark vascular endothelial cells, and showed that purpurin can be especially useful to label bones under this transgenic background where the green fluorescence channel is occupied (Fig. 2F).

**Fig. 3.**
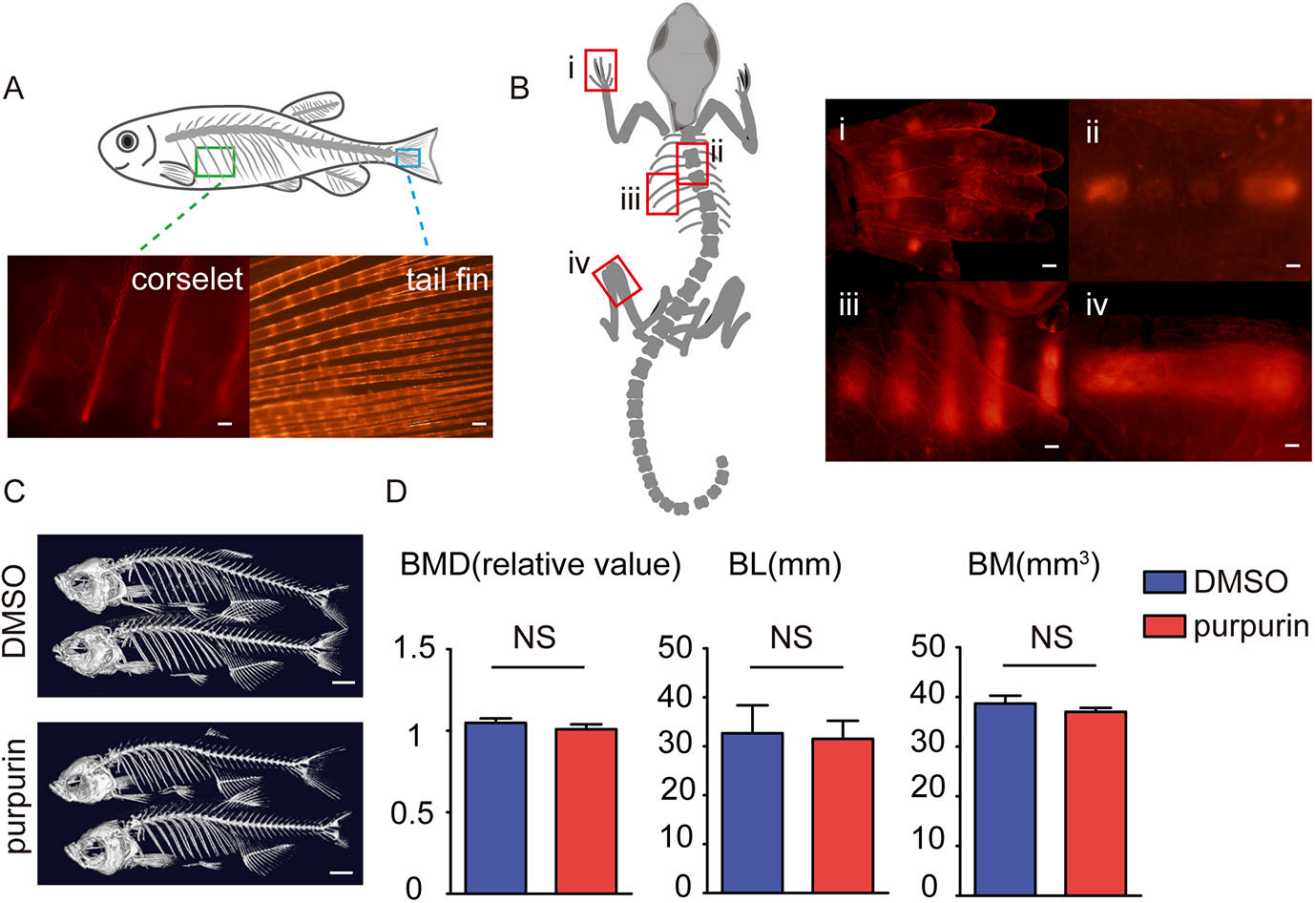
**Purpurin labels bones in adult zebrafish and mice without significantly affecting bone density and mass.** (A) Images of the purpurin-stained corselet and tail fin in an adult zebrafish. Scale bars: 100 μm. (B) Images of the purpurin-stained digital bone (i), spine (ii), rib (iii) and femur (iv) of a P3 mouse. Scale bars: 300 μm. (C) Three-dimensional bone reconstruction of micro-CT scans on the adult zebrafish treated with DMSO and purpurin for 3 weeks. Scale bars: 1 cm. (D) The statistical results of bone mineral density (BMD; *n*=12), body length (BL; *n*=12) and bone mass (BM; *n*=12). Values are means±s.e.m., with differences determined by Student's *t*-test. NS, not significant.

To further assess the potential applications of purpurin as a fluorescent bone dye, staining was performed in live adult zebrafish and postnatal mice. In live adult zebrafish, the red fluorescence was broadly detected in all bone structures including corselet and tail fin 48 h after purpurin treatment (20 μM) (Fig. 3A). In postnatal mice, purpurin (100 μM) was mixed with milk to nurse P0 litters, and the fluorescence was observed around the rib, thoracic vertebra, digital bone and femur after 3 days of constant feeding (Fig. 3B). These results reveal that purpurin is a feasible reagent to visually trace vertebrate and mammalian bone development *in vivo*.

To better investigate the long-term effects of purpurin on bone conditions in living animals, we treated adult zebrafish with purpurin (20 μM) for 3 weeks, and examined the bones in micro-computed tomography (micro-CT). The micro-CT results showed that the bone mineral density (BMD), body length (BL) and bone mass (BM) of the purpurin-treated zebrafish all displayed no significant change (Fig. 3C,D), implying that purpurin can be used to facilitate time-elapse recordings of processes like bone deformation, osteoporosis and bone fracture healing in vertebrates, without affecting the bone properties.

### Epirubicin is hepatotropic and performs better than doxorubicin in suppressing the hyperplasia induced by oncogene over-expression

Several DNA-targeted chemotherapeutic compounds contain fluorophores; doxorubicin and epirubicin were selected in our tissue-specific distribution screening for their strong fluorescence. Doxorubicin was commonly used in the treatment of a wide range of tumours, including blood cancers, solid cancers and soft tissue sarcomas (Tacar et al., 2013). Epirubicin was often used in the treatment of breast cancer, ovarian cancer, gastric cancer, lung cancer and lymphomas (Nitz et al., 2014; Dieci et al., 2015; Petrioli et al., 2016) and caused fewer side-effects (Minotti et al., 2004). Both drugs aimed to block DNA synthesis and were used as a component of several chemotherapy regimens targeting cancer cells in the kidney, lung, heart and liver (Shin et al., 2013; Suarasan et al., 2016).

**Fig. 4.**
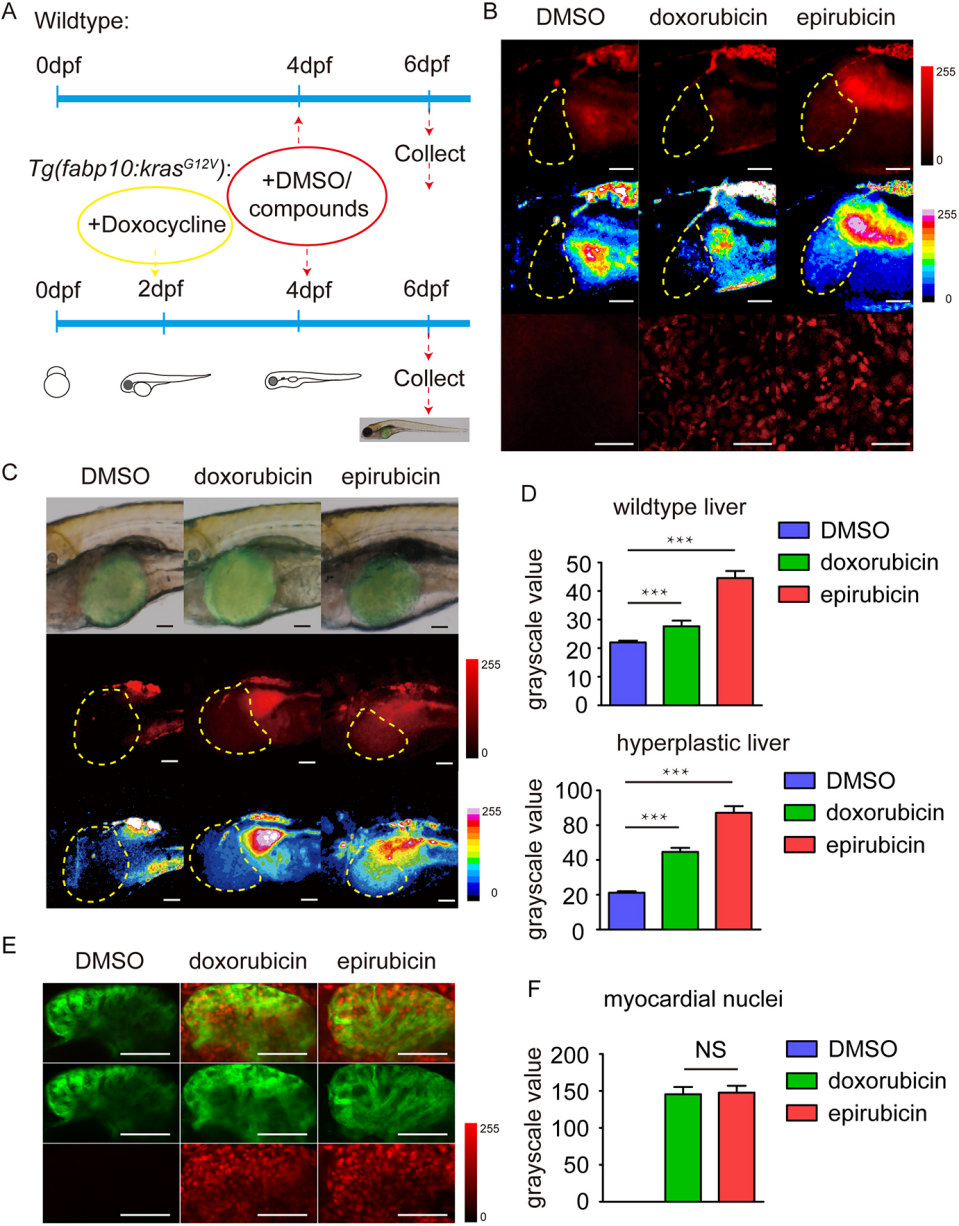
**Distributions of epirubicin and doxorubicin in zebrafish livers and hearts.** (A) The procedures for adding compounds and chemical inducers in wild-type and transgenic zebrafish larvae. (B) Doxorubicin and epirubicin in wild-type zebrafish liver. The top-row panels are the original images taken using the microscope under the red fluorescence channel. The middle-row panels are the images taken with 16 pseudocolours applied to distinguish different densities of the compounds. The bottom-row panels are the confocal images taken to show the nuclear distributions of the compounds in the liver. Scale bars: 50 μm. (C) Doxorubicin and epirubicin in the tumourigenic zebrafish liver with *kras^G12V^* over-expression. The top-row panels are the merged images of the bright-field and green fluorescent channel. The middle-row panels are the original images of the red fluorescent channel. The bottom-row panels are the images with 16 pseudocolours applied to distinguish different densities of the compounds. Scale bars: 50 μm. (D) Statistics of the fluorescence intensity values of wild-type and hyperplastic livers from DMSO-, doxorubicin- and epirubicin-treated zebrafish larvae (*n*=16). The values are means±s.e.m., with statistical differences being determined by one-way ANOVA, ***P<0.001. (E) Doxorubicin and epirubicin in wild-type zebrafish hearts. The green channel displayed the images of *Tg(myl7:EGFP)* zebrafish hearts at 6 dpf, and the red channel displayed doxorubicin or epirubicin in cells. Scale bars: 50 μm. (F) Statistics of the red fluorescence intensities in myocardial nuclei from DMSO-, doxorubicin- and epirubicin-treated zebrafish larvae (*n*=16), means±s.e.m., with statistical differences being determined by one-way ANOVA. NS, no significant changes.

**Fig. 5.**
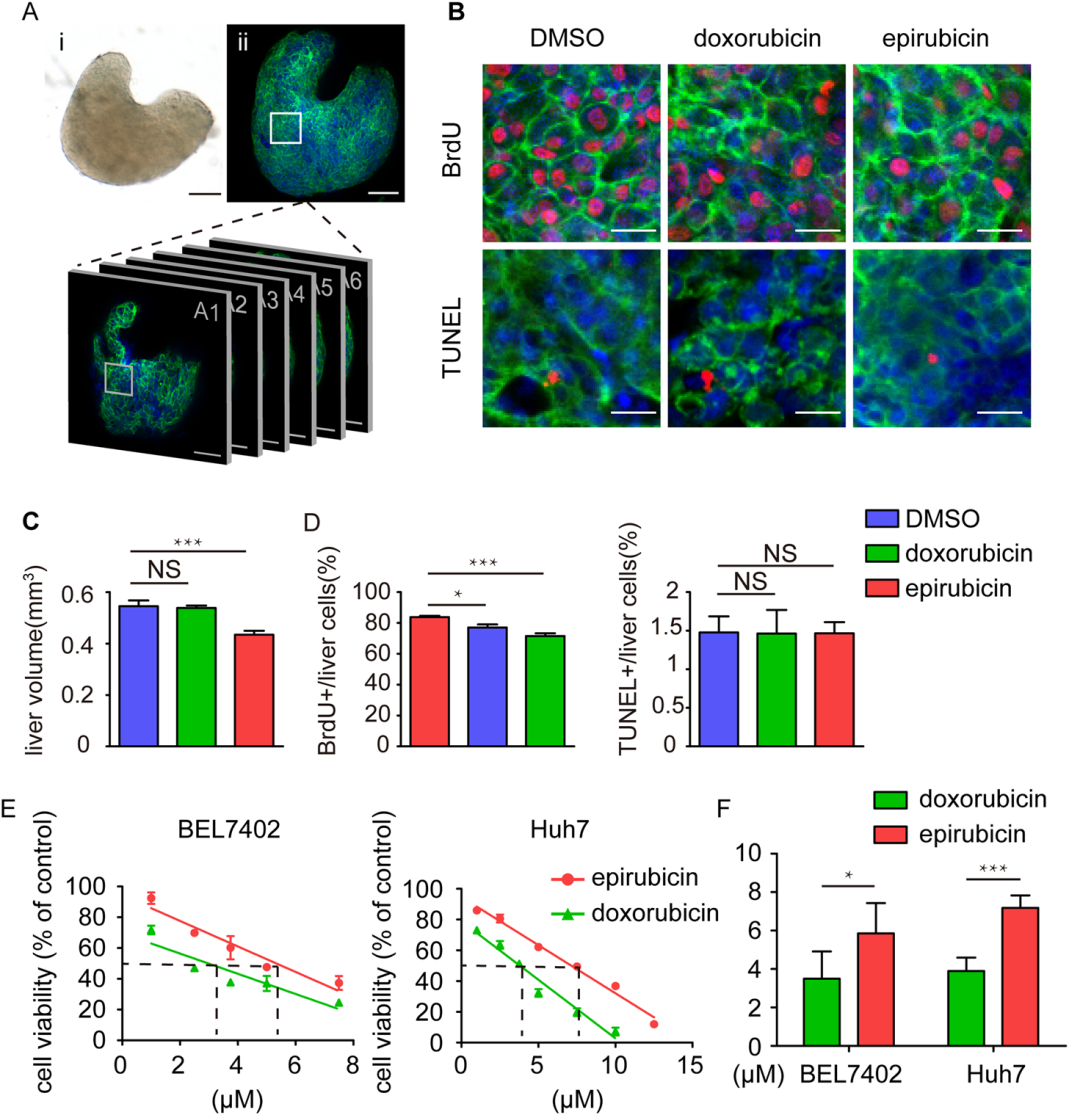
**Effects of doxorubicin and epirubicin on proliferation and apoptosis in the hyperplastic livers of transgenic zebrafish larvae.** (A) An example of the whole liver after dissection in both bright-field and fluorescent images. Scale bars: 50 μm. (B) Representative images of BrdU and TUNEL staining in the DMSO-, doxorubicin- and epirubicin-treated zebrafish liver with *EGFP-kras^G12V^* over-expression. Scale bars: 10 μm. (C) Statistics of the DMSO-, doxorubicin- and epirubicin-treated liver size from the transgenic larvae (*n*=16). The values are means±s.e.m., with statistical differences being determined by one-way ANOVA, ***P<0.001; NS, not significant. (D) Statistics of BrdU and TUNEL labelling in the DMSO-, doxorubicin- and epirubicin-treated zebrafish liver with *EGFP-kras^G12V^* over-expression (*n*=16). The values are means±s.e.m., with statistical differences being determined by one-way ANOVA; *P<0.05, ***P<0.001; NS, not significant. (E) The cytotoxicity of doxorubicin and epirubicin in the liver cancer cells BEL7402 and Huh7 using the MTT assay. The dashed lines are the IC_50_ values for each compound. (F) The statistical results of the IC_50_ of doxorubicin and epirubicin in the liver cancer cells BEL7402 and Huh7. The values are means±s.e.m., with statistical differences being determined by Student's *t*-test; *P<0.05, ***P<0.001.

Interestingly, although both drugs were observed to accumulate in the nucleus of cells around the whole larvae, the whole-mount images showed that the epirubicin-treated larvae bore relatively brighter fluorescence in the liver than the doxorubicin-treated larvae at 6 dpf, indicating that epirubicin maintained a higher concentration in the liver than doxorubicin (Fig. 4B,D). To further confirm the results under tumour-like conditions, we employed a transgenic zebrafish model *Tg(fabp10:rtTA2s-M2; TRE2:EGFP-kras^G12V^)* (Yan et al., 2015), in which *kras^G12V^* expression and the hepatic hyperplasia phenotype can be chemically induced by adding doxycycline to the zebrafish liver from 2 dpf to 6 dpf (Fig. 4A). Doxorubicin and epirubicin (10 μM) were then added to the induced transgenic larvae at 4 dpf, and their fluorescence distribution patterns at 6 dpf suggested that epirubicin was more inclined to accumulate in the tumourigenic zebrafish livers than doxorubicin (Fig. 4C,D). As cardiotoxicity is one of the most severe side-effects of doxorubicin, we also examined the distributions of doxorubicin and epirubicin in zebrafish hearts at 6 dpf. The *Tg(myl7:EGFP)* zebrafish line was used to help label myocardial cells with green fluorescence, and confocal optical sectioning was used to avoid background autofluorescence. We measured the red fluorescence intensities in myocardial nuclei from larvae treated with doxorubicin and epirubicin, and detected no significant difference between them (Fig. 4E,F). To investigate the tumour-repressing functions of doxorubicin and epirubicin in the zebrafish model of hepatic hyperplasia, we first measured the liver size of the induced transgenic larvae treated with and without drugs. Only epirubicin was observed to significantly reduce liver size, and doxorubicin at the same concentration had no significant effect (Fig. 5C). BrdU assays were then performed to evaluate the proliferation of hepatocytes, and the results suggested that epirubicin significantly inhibited their proliferation, while doxorubicin at the same concentration showed moderate effects (Fig. 5B,D). As apoptosis was previously reported to increase in cultured hepatic cells after treatment with epirubicin and doxorubicin (Henninger et al., 2012; Du et al., 2013), TUNEL staining assays were also performed to assess apoptosis of hepatocytes. Unlike previous reports, our results indicated that there was no significant difference in hepatocyte cell death incidences after treatment with either drug, probably due to the shorter experimental period in our assays (Fig. 5B,D).

As epirubicin and doxorubicin were reported to have different cytotoxic effects, we could not rule out the possibility that the liver-specific anti-tumour effects of epirubicin over doxorubicin may be caused by differences in bioactivities. Here we tested the cytotoxic effects of both compounds in two human HCC cell lines, BEL7402 and Huh7 (Fig. 5E,F). The half-maximal inhibitory concentration (IC_50_) of doxorubicin for BEL7402 after 24 h incubation was 3.1 μM, and the IC_50_ of epirubicin was 5.5 μM. For Huh7, the IC_50_ of doxorubicin was 3.8 μM, and the IC_50_ of epirubicin was 7.1 μM. These data suggest that doxorubicin was more toxic to hepatic tumour cells than epirubicin, and indirectly supported the idea that the liver-specific distribution of epirubicin may contribute to its greater inhibitory effect on the *kras^G12V^*-insulted hepatocyte proliferation.

## DISCUSSION

Investigating the tissue-specific distribution of a certain compound can reveal a lot of information, including the potential side-effects or the possibilities for drug repositioning (Esch et al., 2015). Zebrafish larvae are convenient and cost-effective tools to perform high-throughput fluorescence-based screening, and here we described the biodistribution patterns of 15 compounds as a preliminary resource to the zebrafish community. Our studies identified several small molecules, for the first time, as feasible red fluorescent dyes to label collagens and bone structures in live vertebrates, and also implied that the tissue-specific distribution patterns of compounds in zebrafish larvae can provide us with direct visual information related to the clinical pharmacokinetic indexes, and may help direct the development of novel drugs and therapies.

Compounds with similar structures may share similar distribution patterns. In this paper, we identified three red fluorescent dyes in the 9,10-anthraquinone family of compounds (lucidin, purpurin and 3-hydroxy-morindone) that can be used to stain and trace developing bones in live vertebrates. These dyes can be applied directly to live animals with significant advantages over the traditional Alizarin Red-staining on fixed samples, and even the green fluorescent bone dye calcein (Salvaggio et al., 2016), as these compounds emit at a longer wavelength.

In general, the 9,10-anthraquinone compound family exhibits a variety of light-active properties under UVA or fluorescent light exposure (Zhuo and Sun, 2013). For example, 1,4-dihydroxyanthraquinone 1 and aloe-emodin, which are also 9,10-anthraquinones, exhibit fluorescence emission in the yellow channel and were used as a fluorescent zinc probe *in vivo* (Francis et al., 2014; Sinha et al., 2015). Moreover, 9,10-anthraquinones can also be used to tag other molecules, e.g. a novel compound composed of porphyrin and a 9,10-anthraquinone derivative was synthesized to evaluate the anti-proliferation activity in HeLa cells (Yang et al., 2016). Interestingly, the three 9,10-anthraquinones found in this study were observed to be osteophilic. In future studies, it may be worth pursuing whether the 9,10-anthraquinones can be used as a bone-seeking tag to bring other molecules or drugs to bones for a therapeutic purpose after chemical conjugation. In addition, the safety of these compounds as drugs remains debatable. Although purpurin showed no significant effects on bone mass and density in our 3-week experiment, it was reported that purpurin inhibited larval angiogenesis through inhibiting adipocyte-derived leucine aminopeptidase (A-LAP) in zebrafish at 10 μM (Park et al., 2014). Moreover, the natural source of these compounds, *Rubia tinctorum*, was widely used as a traditional medicine against kidney stones (Westendorf et al., 1998), and both kidney stones and bones contain calcium salts, indicating a potential risk of osteolytic destruction.

Clinically, epirubicin was regarded as a better chemotherapeutic option than doxorubicin, given its lower cardiotoxicity (Minotti et al., 2004). Our results demonstrated that epirubicin is more effective in repressing hyperplasia and ectopic proliferation in zebrafish hepatic cells possessing increased oncogene expression. This is probably due to epirubicin being more hepatotropic. Interestingly, it has also been reported that epirubicin extensively accumulates in rat liver and is excreted in the bile (Shin et al., 2013, 2014), which is the same phenomenon that we observed in zebrafish larvae. This suggests that drug distributions in zebrafish can partially reflect the pharmacokinetics in mammals. Given the similarity between zebrafish and mammals, it may be worthwhile to conduct comparative studies of the distribution patterns of all chemotherapeutic drugs in zebrafish. This information may be used to optimize chemotherapy regimens for different types of cancers, after being confirmed in pharmacokinetics analyses. Our study suggests that the transparent zebrafish larva is a powerful and convenient platform for identifying tissue-specific fluorescent dyes, assessing their side-effects, predicting the novel applications of these compounds, and evaluating the pharmacokinetics of fluorescent or fluorescence-label compounds. A systematic preliminary trial on zebrafish larvae may prove extremely valuable to the optimization of combination chemotherapies.

## MATERIALS AND METHODS

### Zebrafish lines and mice

Zebrafish embryos, larvae and adult fish were raised under standard laboratory conditions at 28.5°C. AB wild-type strain, *Tg(flk1:EGFP)* (Jin et al., 2005), *Tg(myl7:EGFP)* (Aguirre et al., 2014) and *Tg(fabp10:rtTA2s-M2; TRE2:EGFP-kras^G12V^)* (Yan et al., 2015) were used in the experiments. The *Tg(fabp10:rtTA2s-M2; TRE2:EGFP-kras^G12V^)* line was a generous gift from Prof. Gong's Laboratory at the National University of Singapore. To over-express oncogene *kras^G12V^* in hepatocytes, transgenic zebrafish were bathed in E3 medium with 60 μg ml^−1^ doxycycline (Sigma D9691) from 2 dpf to 6 dpf. C57/BL6 mice were used for the study involving mouse litters. All studies involving animal manipulations were approved by the Fudan University Shanghai Medical School Animal Care and Use Committee and followed the National Institutes of Health guidelines for the care and use of animals.

### Small-molecule compounds

Small-molecule compound candidates were selected from the Fudan MolMed-Selleck Compound Library (FMSCL). Stock concentrations of each compound are listed in the Table S1, and the working solutions were diluted in E3 medium at 1:1000 from the stock solution. Zebrafish larvae and adults were directly treated with the working solution, and the juvenile mice were treated by feeding them milk containing the compounds (1:100). Zebrafish larvae were treated with 20 μM dexamethasone (Sigma D1756) from 2 dpf to 6 dpf to help identify developmental bone defects.

### Imaging and image processing

The live zebrafish larvae including 3, 6 and 14 dpf zebrafish in the fluorescent channel were mounted in 3% methylcellulose (Sigma M0512) containing 200 mg l^−1^ tricaine (Sigma A5040) before imaging under a fluorescent dissecting scope (Olympus DP73). The fixed zebrafish larvae after Alizarin Red and Alcian Blue skeletal staining were mounted in 80% glycerol (Sigma G9012) before imaging under a fluorescent dissecting scope. Adult zebrafish were anesthetized in 0.2% tricaine and then killed by incubation in ice water for 15 min. Mice were killed by cervical dislocation. Both adult zebrafish and mice were fixed in 4% paraformaldehyde (PFA) overnight before imaging. Confocal images were obtained using a Leica TCS SP8 confocal microscope. Cell numbers and percentages were manually counted from the confocal optical sections, and pseudo-colours of fluorescence intensities were processed via Image J, an open-source software.

### Collagen I antibody staining

The swim bladders of adult zebrafish were dissected on ice. The whole swim bladders were washed with phosphate buffered solution (PBS) three times and then fixed in 4% PFA for 4 h at room temperature. The samples were washed with PBS again three times and blocked in PBS with 0.1% Triton X-100 and 10% normal goat serum (PBT) for 1 h at room temperature. The samples were then incubated with collagen I antibody (Boster BA0325 1:250) in PBT overnight. Goat anti-rabbit Alexa 488 (Jackson ImmunoResearch 115-035-003 1:500) was used as the secondary antibody, and after 4 h, the sample was washed three times in PBS before mounting and imaging.

### Whole-mount Alizarin Red and Alcian Blue skeletal staining

The 14 dpf zebrafish were fixed in 4% PFA for 4 h, and dehydrated with 50% ethanol for 10 min. After being stained with acid-free 0.5% Alizarin Red (Sigma A5533) and 0.02% Alcian Blue (Sigma A5268) overnight, the samples were washed with distilled water and bleached with 3% H_2_O_2_–2% KOH. The samples were then washed with 20% glycerol–0.25% KOH for 30 min, followed by 50% glycerol–0.25% KOH for 2 h. All the above procedures were performed at room temperature.

### Calcein staining

Calcein solution (0.2%) was prepared by dissolving 2 g of calcein powder (TCI C0004) in 1 litre of deionized water (pH 7.4). Zebrafish larvae were treated with 0.04% calcein in E3 medium for 10 min, washed in E3 medium three times, and rinsed in E3 medium for 10 min before imaging.

### BrdU immunohistochemistry and TUNEL staining

Zebrafish larvae were incubated in 10 mM BrdU (Sigma B5002) for 12 h before fixing. After the incubation, the larvae were immediately washed six times in E3 medium for 1 h. The samples were then fixed in 4% PFA for 4 h at room temperature. After 2 h, we dissected the liver from the whole larvae and immunostaining was performed as described previously (Wang et al., 2012) using the following antibodies: BrdU mouse antibody Alexa Fluor 647 (Thermo Fisher, B35133, 1:2000) and Hoechst 33342 (Thermo Fisher, H3570, 1:5000). TUNEL assays were carried out using the ApopTag Red *in situ* Apoptosis Detection Kit (Millipore, S7165).

### Micro-computed tomography

Whole zebrafish were scanned using a Scanco μCT 40 desktop cone-beam micro-CT scanner (Quantum FX, PerkinElmer, Hopkinton, MA, USA) set at 70 kV and 114 μA. Zebrafish were aligned and embedded in 1% agarose, followed by scanning in a 36-mm diameter sample holder at a resolution of 72 μm. Caliper microCT Analysis Tools Analyze (Quantum FX, PerkinElmer) was used to process the raw data.

### Cytotoxicity assay

Cytotoxic effects of epirubicin and doxorubicin were evaluated by the3-(4,5-dimethylthiazol-2-yl)-2,5-diphenyltetrazolium bromide (MTT) assay (Beytime, C0009). HCC cell lines BEL7402 and Huh7 were obtained from the Cell Bank of Type Culture Collection of Chinese Academy of Sciences, Shanghai Institute of Cell Biology, Chinese Academy of Sciences. Cells were cultured in Dulbecco's modified Eagle's medium (DMEM) (HyClone SH30022.01) supplemented with 10% fetal bovine serum (FBS) (Gibco 10099133) at 37°C with 10% CO_2_. Both cell lines were seeded in 96-well plates at a density of 1.5×10^5^ cells/well, and were treated with epirubicin or doxorubicin at different concentrations (ranging from 1 to 15 μM) for 24 h. Cells were then washed once in PBS and incubated with 0.5 mg ml^−1^ MTT in serum-free medium for 4 h at 37°C. Afterwards, the medium was removed, and 100 μl dimethyl sulfoxide (DMSO) was added. The absorbance was read and analysed at 550 nm via a microplate reader (Multiskan Spectrum, Thermo Scientific).

### Statistical analyses

All Student's *t*-tests and one-way ANOVAs were performed in GraphPad Prism 6 (GraphPad Software Inc.) and *P*<0.05 was considered significant.
